# Supplementary material for: Decoding Immuno-Competence: A Novel Analysis of Complete Blood Cell Count Data in COVID-19 Outcomes
Source: Biomedicines. 2024 Apr 15;12(4):871. doi: 10.3390/biomedicines12040871 (PMC11048687; doi:10.3390/biomedicines12040871)
Supplement: Supplementary file 1 [file biomedicines-12-00871-s001.zip › biomedicines-2891345-supplementary.pdf]

Table S1

Table S1. Longitudinal assessments of 101 COVID-19 patients (n=291)

Obs	ID #	Day	Outcome	PCT	IL-6	Ferrit	C-RP	LDH	D-Dimer	Hb	Hemat	WBC	Neutrophil	Monocyte	Lymphocyte	Protective profiles
1	1	1	Survivor	0.36	22	747	202	355	923	13	37.6	8.1	81.4	7.0	11.5	Other
2	1	2	Survivor	0.31	22	832	139	383	723	14	40.6	8.3	83.6	8.0	8.2	Other
3	1	3	Survivor	0.27	10	717	74	347	631	13	35.9	11.4	86.5	5.8	7.5	Other
4	1	4	Survivor	0.18	0	1019	73	413	3989	12	34.4	11.4	88.4	4.4	7.1	Other
5	1	5	Survivor	0.07	0	656	7	248	1204	12	34.2	12.9	91.4	3.2	5.2	Other
6	2	1	Survivor	0.07	36	485	71	122	265	14	42.0	6.5	71.7	12.1	16.1	Other
7	2	2	Survivor	0.07	10	730	156	124	220	13	40.0	6.3	57.4	6.9	35.5	Other
8	2	3	Survivor	0.07	0	601	146	179	410	15	43.8	15.3	57.4	6.9	35.5	Other
9	3	1	Survivor	0.39	27	833	153	317	395	14	44.3	13.2	93.3	4.1	2.5	Other
10	3	2	Survivor	0.26	7	833	172	313	471	13	40.4	10.9	93.5	2.3	4.1	II
11	3	3	Survivor	0.17	8	*	73	321	743	13	40.2	12.1	88.4	6.0	5.4	Other
12	4	1	Survivor	0.16	7	530	229	535	1337	13	42.6	7.2	83.9	4.3	11.7	Other
13	4	2	Survivor	0.13	9	506	119	755	1253	12	40.2	6.0	83.4	4.0	12.5	Other
14	4	3	Survivor	0.13	61	421	86	425	1237	13	42.0	8.9	84.9	5.9	9.0	Other
15	4	4	Survivor	0.10	*	332	83	395	1576	12	42.0	8.9	78.5	6.4	14.9	Other
16	5	1	Survivor	0.10	82	2961	118	451	1201	14	41.8	7.9	79.5	11.7	8.7	Other
17	5	2	Survivor	0.10	82	2642	118	364	798	14	40.9	6.8	76.4	11.7	11.7	Other
18	5	3	Survivor	0.10	40	1183	135	272	546	11	35.3	7.3	76.1	12.1	11.7	Other
19	6	1	Survivor	0.30	314	1078	108	277	1019	16	47.6	9.0	88.0	4.8	7.0	Other
20	6	2	Survivor	0.33	314	1311	168	277	678	13	42.9	5.5	87.6	5.8	6.4	Other
21	6	3	Survivor	0.28	380	1151	109	382	783	14	42.6	6.3	82.7	8.5	8.7	Other
22	6	4	Survivor	0.20	6	693	8	170	561	13	39.4	6.3	69.1	11.1	19.6	Other
23	7	1	Survivor	0.09	18	175	9	336	672	13	40.0	5.4	65.9	15.4	18.6	Other
24	7	2	Survivor	0.09	18	175	9	336	672	12	37.5	4.6	69.5	15.6	14.8	Other
25	7	3	Survivor	0.09	18	126	6	205	539	10	31.6	2.6	52.3	25.8	21.8	Other
26	8	1	Survivor	0.11	30	339	83	297	504	10	36.9	4.5	57.2	8.7	33.9	Other
27	8	2	Survivor	0.13	*	547	122	262	484	11	33.1	9.1	79.6	8.5	11.8	Other
28	8	3	Survivor	*	*	*	*	*	*	11	34.9	9.8	82.9	7.5	9.4	Other
29	9	1	Survivor	*	24	1500	4	258	228	15	44.4	7.7	70.4	12.7	16.8	Other
30	9	2	Survivor	0.06	24	1442	6	261	282	13	40.3	5.2	55.8	10.7	33.3	Other
31	9	3	Survivor	0.06	*	1878	10	306	277	13	41.5	6.3	58.2	13.5	28.1	Other
32	10	1	Survivor	0.13	26	53	5	212	404	8	27.2	8.9	85.6	8.1	6.2	Other
33	10	2	Survivor	0.13	26	53	5	212	404	7	24.7	7.7	92.2	3.0	4.7	Other
34	10	3	Survivor	0.13	26	55	7	249	650	7	24.7	8.1	87.7	8.5	3.7	Other
35	10	4	Survivor	*	*	21	*	255	703	7	22.6	11.4	85.6	9.7	4.6	Other
36	11	1	Survivor	0.07	11	328	11	161	220	14	42.7	6.7	78.0	10.3	11.6	Other
37	11	2	Survivor	0.06	11	363	7	163	*	14	44.2	7.4	71.6	10.4	17.9	Other
38	11	3	Survivor	0.06	*	363	7	163	*	14	44.0	7.1	71.7	8.4	19.7	Other
39	12	1	Survivor	*	33	115	11	194	979	7	24.6	4.0	42.5	20.5	36.8	III
40	12	2	Survivor	*	33	115	11	194	1341	7	24.6	4.0	42.5	20.5	36.8	III
41	12	3	Survivor	*	33	199	20	213	844	7	24.3	3.5	46.3	17.4	36.	III
42	13	1	Survivor	0.14	43	38	133	236	917	9	29.1	7.6	69.5	13.0	17.3	Other
43	13	2	Survivor	0.14	43	38	133	236	917	8	29.1	4.3	43.0	15.3	41.5	III
44	13	3	Survivor	0.16	*	47	82	194	746	8	27.1	6.0	47.4	13.2	39.3	III
45	14	1	Survivor	0.11	24	788	12	242	285	14	43.0	6.0	76.7	7.2	16.0	Other
46	14	2	Survivor	0.11	24	788	242	114	285	13	38.7	5.0	71.0	8.6	20.3	Other
47	14	3	Survivor	0.11	24	779	10	255	249	13	40.6	4.4	48.7	13.2	37.9	III
48	15	1	Survivor	0.13	53	154	203	21	1926	12	36.9	4.4	63.4	15.1	21.3	Other
49	15	2	Survivor	0.13	53	154	203	22	1926	11	35.7	4.0	55.7	20.9	23.2	Other
50	15	3	Survivor	0.13	212.	153	203	22	1211	11	36.2	5.1	60.4	19.2	20.3	Other

Table S1 - cont'd

Obs.	ID #	Day	Outcome	PCT	IL-6	Ferrit	C-RP	LDH	D-Dimer	Hb	Hemat	WBC	Neutrophil	Monocyte	Lymphocyte	Protective profiles
51	16	1	Survivor	25.42	3543	1404	104	340	574	16	47	4.40	89.8	3.1	7.0	Other
52	16	2	Survivor	18.57	37	1764	174	404	326	16	48	8.90	85.1	2.7	12.0	Other
53	16	3	Survivor	12.38	17	1139	132	415	422	15	46	8.90	92.8	2.8	4.3	II
54	17	1	Survivor	0.07	23	470	10	283	505	13	42	3.60	89.9	2.7	7.3	Other
55	17	2	Survivor	0.07	25	562	13	264	382	13	42	3.90	86.2	4.6	9.0	Other
56	17	3	Survivor	0.07	25	837	43	346	419	12	39	4.10	77.7	4.9	17.3	Other
57	18	1	Non-survivor	0.16	332	675	85	573	873	13	41	4.90	72.3	6.1	21.4	Other
58	18	2	Non-survivor	0.16	332	833	106	573	844	14	44	5.60	65.7	9.6	24.6	Other
59	18	3	Non-survivor	0.00	0	673	70	855	928	12	37	7.60	75.8	6.4	17.7	Other
60	19	1	Non-survivor	0.22	39	534	92	1102	13932	12	37	11.10	66.1	8.1	25.7	Other
61	19	2	Non-survivor	0.20	39	543	141	1111	6997	11	35	7.40	67.8	7.8	24.2	Other
62	19	3	Non-survivor	0.22	4500	645	289	834	3230	12	37	8.40	86.2	4.6	9.1	Other
63	19	4	Non-survivor	0.63	0	563	22	829	4283	11	33	14.00	86.0	5.8	8.0	Other
64	19	5	Non-survivor	0.37	0	0	35	344	6327	10	31	13.80	83.6	7.5	8.8	Other
65	20	1	Non-survivor	0.13	481	385	*	341	2414	12	37	13.80	90.2	1.9	7.8	Other
66	20	2	Non-survivor	2.05	481	428	*	299	3850	11	36	12.10	85.9	3.6	10.4	Other
67	20	3	Non-survivor	2.05	0	852	375	484	3836	11	35	12.80	85.7	5.3	8.9	Other
68	20	4	Non-survivor	0.00	0	745	169	319	32908	10	30	13.00	84.8	8.3	6.8	Other
69	21	1	Non-survivor	0.64	462	2166	204	755	667	14	42	7.60	80.5	7.4	11.9	II
70	21	2	Non-survivor	0.00	525	3068	124	971	489	11	35	11.70	78.3	9.1	12.5	Other
71	22	1	Survivor	1.12	282	1403	278	423	1260	13	41	8.60	51.2	9.4	39.2	II
72	22	2	Survivor	0.75	300	1779	324	552	1156	14	41	7.70	44.3	10.1	45.5	Other
73	22	3	Survivor	0.25	265	1797	292	477	2199	13	39	7.40	47.9	6.7	45.3	Other
74	23	1	Non-survivor	1.21	20	149	81	286	1829	9	28	13.70	44.6	12.6	42.6	Other
75	23	2	Non-survivor	0.00	0	113	65	268	1672	7	23	13.80	52.6	7.5	39.	Other
76	23	3	Non-survivor	0.00	0	257	54	207	3709	8	26	15.40	51.0	9.0	39.9	Other
77	24	1	Non-survivor	0.36	128	2868	138	350	367	15	46	9.20	65.5	9.7	24.6	Other
78	24	2	Non-survivor	0.26	154	2691	164	420	393	14	42	9.30	75.2	5.6	19.1	Other
79	24	3	Non-survivor	0.18	*	2588	82	425	390	14	43	9.00	76.9	6.6	16.3	Other
80	25	1	Survivor	0.18	154	448	4	488	5012	12	36	8.20	60.5	6.4	33.0	Other
81	25	2	Survivor	0.17	103	376	3	398	4919	12	37	7.10	87.9	4.2	7.8	Other
82	25	3	Survivor	0.17	55	95	7	302	2237	12	36	9.00	70.8	5.9	23.2	Other
83	26	1	Survivor	0.09	53	271	113	313	1146	11	35	8.30	78.3	7.6	13.9	Other
84	26	2	Survivor	0.08	3	230	110	281	1038	12	36	5.60	66.0	11.0	22.8	Other
85	26	3	Survivor	0.07	0	280	45	270	1427	12	37	3.80	67.5	10.6	21.8	Other
86	27	1	Survivor	0.91	175	1001	245	617	1972	13	42	14.40	63.7	11.0	25.1	II
87	27	2	Survivor	0.27	3620	725	214	556	2132	10	33	12.80	84.6	3.1	12.2	II
88	27	3	Survivor	0.16	880	1845	206	623	3264	12	37	14.60	64.5	12.4	23.0	Other
89	28	1	Survivor	0.08	43	245	32	291	736	13	39	5.10	66.5	10.8	22.6	Other
90	28	2	Survivor	0.00	0	226	35	238	361	11	34	4.30	81.8	10.7	7.3	III
91	29	1	Survivor	0.35	12	945	92	575	487	14	42	5.40	87.0	5.7	7.2	Other
92	29	2	Survivor	0.31	4	925	102	459	486	14	40	6.20	87.1	6.5	6.2	Other
93	29	3	Survivor	0.23	0	938	80	336	836	13	39	4.60	76.8	5.9	17.1	Other
94	30	1	Survivor	0.15	50	81	90	420	884	12	40	9.20	63.9	10.6	25.4	Other
95	30	2	Survivor	0.12	157	79	101	451	779	12	39	5.40	64.5	9.3	26.1	Other
96	30	3	Survivor	0.31	756	110	68	629	858	11	36	6.50	75.3	6.8	17.8	Other
97	31	1	Non-survivor	0.01	72	996	99	733	699	15	46	6.50	74.6	6.4	18.8	Other
98	31	2	Non-survivor	0.08	431	744	96	756	895	14	44	3.40	65.6	5.6	28.6	Other
99	31	3	Non-survivor	0.00	4500	696	46	889	992	15	47	14.60	59.0	6.9	34.0	Other
100	32	1	Survivor	0.45	221	253	173	223	1066	13	39	10.50	67.3	7.5	25.1	Other

Table S1 - cont'd

Obs.	ID #	Day	Outcome	PCT	IL-6	Ferrit	C-RP	LDH	D-Dimer	Hb	Hemat	WBC	Neutrophil	Monocyte	Lymphocyte	Protective profiles
101	32	2	Survivor	0.74	171	295	175	245	1356	12	36	10.5	89.8	3.1	7.0	Other
102	32	3	Survivor	3.21	220	737	199	391	890	12	37	8.5	85.1	2.7	12.0	Other
103	33	1	Survivor	0.38	69	997	144	549	615	13	373	9.4	92.8	2.8	4.3	Other
104	33	2	Survivor	0.33	69	1058	141	392	503	12	35	5.9	89.9	2.7	7.3	Other
105	33	3	Survivor	0.07	69	567	68	319	562	12	3	8.3	86.2	4.6	9.0	Other
106	34	1	Survivor	0.41	40	518	118	367	329	14	41	5.6	77.7	4.9	17.3	Other
107	34	2	Survivor	0.30	16	496	116	352	287	13	40	5.7	72.3	6.1	21.4	Other
108	34	3	Survivor	0.30	16	537	127	357	338	13	40	4.1	65.7	9.6	24.6	Other
109	35	1	Survivor	4.56	15	110	58	325	708	10	32	4.1	75.8	6.4	17.7	Other
110	35	2	Survivor	0.46	15	92	56	389	708	10	3	6.0	66.1	8.1	25.7	Other
111	35	3	Survivor	0.21	15	72	28	368	708	10	32	7.9	67.8	7.8	24.2	Other
112	36	1	Survivor	1.52	857	545	171	749	522	14	42	5.7	86.2	4.6	9.1	Other
113	36	2	Survivor	1.36	182	592	196	840	329	14	46	5.1	86.0	5.8	8.0	Other
114	36	3	Survivor	0.22	24	630	137	649	340	13	40	4.7	83.6	7.	8.8	Other
115	37	1	Survivor	0.23	322	1957	125	658	975	13	41	6.9	90.2	1.9	7.8	II
116	37	2	Survivor	0.42	91	1778	186	715	707	12	38	7.3	85.9	3.6	10.4	Other
117	37	3	Survivor	0.30	16	1828	113	608	787	12	38	8.0	85.7	5.3	8.9	Other
118	38	1	Survivor	0.14	54	617	103	493	432	13	40	9.5	84.8	8.3	6.8	Other
119	38	2	Survivor	0.06	111	460	85	419	409	13	39	6.2	80.5	7.4	11.9	Other
120	38	3	Survivor	0.00	8	464	50	397	471	13	70	7.0	78.3	9.1	12.5	Other
121	39	1	Survivor	0.00	5	39	0	227	5943	13	41	5.4	51.2	9.4	39.2	III
122	39	2	Survivor	0.00	5	185	3	252	4820	13	41	5.1	44.3	10.1	45.5	III
123	39	3	Survivor	0.00	5	249	4	258	1429	13	41	4.9	47.9	6.7	45.3	III
124	40	1	Survivor	0.06	24	59	6	229	3449	11	36	1.5	44.6	12.6	42.6	III
125	40	2	Survivor	0.06	21	66	7	213	240	11	35	2.4	52.6	7.5	39.8	III
126	40	3	Survivor	0.06	20	73	5	292	380	12	39	2.5	51.0	9.0	39.9	III
127	41	1	Survivor	*	*	*	*	*	*	12	41	8.1	65.5	9.7	24.6	Other
128	42	1	Survivor	0.07	8	168	26	159	1162	13	41	3.7	75.2	5.6	19.1	Other
129	42	2	Survivor	0.08	17	215	28	154	1299	12	39	3.5	76.9	6.6	16.3	Other
130	42	3	Survivor	0.00	6	226	30	189	872	12	40	2.8	60.5	6.4	33.0	Other
131	43	1	Survivor	0.17	94	332	82	327	1031	12	39	8.1	87.9	4.2	7.8	Other
132	43	2	Survivor	0.51	3	358	70	279	684	10	31	5.1	70.8	5.9	23.2	Other
133	43	3	Survivor	0.23	0	473	52	359	534	11	35	7.2	78.3	7.6	13.9	Other
134	44	1	Survivor	0.06	17	13	11	330	429	9	31	5.5	66.0	11.0	22.8	Other
135	44	2	Survivor	0.07	17	13	7	240	404	8	31	5.5	67.5	10.6	21.8	Other
136	44	3	Survivor	0.07	17	13	5	200	475	9	32	7.9	63.7	11.0	25.1	Other
137	45	1	Survivor	0.20	87	638	205	290	481	13.	41	10.1	84.6	3.1	12.2	Other
138	45	2	Survivor	0.19	25	888	215	275	1652	13	41	4.0	64.5	12.4	23.0	Other
139	45	3	Survivor	0.15	25	570	127	269	885	13	41	9.7	66.5	10.8	22.6	Other
140	46	1	Survivor	0.11	106	746	88	476	3956	14	42	8.9	81.8	10.7	7.3	Other
141	46	2	Survivor	0.07	106	631	87	459	3206	13	41	5.5	87.0	5.7	7.2	Other
142	46	3	Survivor	0.06	106	556	27	372	1520	13	39	6.3	87.1	6.5	6.2	Other
143	47	1	Survivor	0.17	30	344	83	461	554	14	36	6.8	76.8	5.9	17.1	Other
144	47	2	Survivor	0.17	16	346	106	423	505	14	44	5.1	63.9	10.6	25.4	Other
145	47	3	Survivor	0.17	7	347	57	330	531	14	45	6.3	64.5	9.3	26.1	Other
146	48	1	Survivor	0.67	71	18	180	590	2066	13	42	7.1	75.3	6.8	17.8	Other
147	48	2	Survivor	0.60	11	305	102	537	1137	12	38	6.5	74.6	6.4	18.8	Other
148	48	3	Survivor	0.40	0	220	69	463	1571	12	37	6.5	65.6	5.6	28.6	Other
149	49	1	Survivor	0.11	*	108	6	*	473	13	41	3.6	59.0	6.9	34.0	Other
150	49	2	Survivor	0.08	*	206	6	*	280	13	38	11.7	67.3	7.5	25.1	Other

Table S1 - cont’d

Obs.	ID #	Day	Outcome	PCT	IL-6	Ferrit	C-RP	LDH	D-Dimer	Hb	Hemat	WBC	Neutrophil	Monocyte	Lymphocyte	Protective profiles
151	50	1	Survivor	0.08	53	621	46	355	803	15	44	10.7	86.2	7.1	6.5	Other
152	50	2	Survivor	0.00	53	651	88	334	624	14	41	6.2	71.3	10.9	17.6	Other
153	50	3	Survivor	0.00	53	616	54	317	520	14	45	4.7	75.5	9.1	15.3	Other
154	51	1	Survivor	0.06	7.70	87.00	28	366	491	14	43	2.3	79.5	3.9	16.5	Other
155	51	2	Survivor	0.07	82	114	22	326	453	13	40	4.1	73.9	5.1	20.9	Other
156	51	3	Survivor	0.07	7	92	22	320	333	12	37	3.6	86.9	4.7	8.3	Other
157	52	1	Survivor	0.27	35	855	92	318	800	14	42	8.1	84.5	6.2	9.1	Other
158	52	2	Survivor	0.20	5	765	42	401	997	14	42	5.9	81.7	6.9	11.2	Other
159	52	3	Survivor	0.15	5	886	101	419	1008	13	41	12.8	90.8	3.7	5.4	Other
160	53	1	Non-survivor	0.76	97	948	208	429	845	11	35	4.9	91.3	2.4	6.1	II
161	53	2	Non-survivor	1.53	108	1223	133	418	1034	12	37	4.6	88.1	4.4	7.4	Other
162	53	3	Non-survivor	1.41	108	834	91	459	1349	12	35	4.0	90.2	3.5	6.2	Other
163	54	1	Survivor	0.08	82	526	140	347	530	15	43	5.3	86.2	5.5	8.2	Other
164	54	2	Survivor	0.08	87	399	174	367	399	15	44	3.2	75.7	7.1	17.1	Other
165	54	3	Survivor	0.08	83	364	88	374	551	14	44	3.8	67.1	12.2	20.5	Other
166	55	1	Survivor	2.54	49	476	83	291	611	16	48	5.3	74.1	5.5	20.3	Other
167	55	2	Survivor	3.66	49	469	112	336	587	14	44	4.2	67.4	7.1	25.3	Other
168	55	3	Survivor	2.32	49	665	27	489	886	14	43	9.5	76.0	6.6	17.2	Other
169	56	1	Survivor	0.06	20	144	71	167	1204	10	36	9.5	73.3	7.5	19.0	Other
170	56	2	Survivor	0.06	20	107	59	167	1011	8	28	9.3	65.2	7.3	27.3	Other
171	57	1	Survivor	0.79	50	1143	15	0	1079	13	38	7.8	70.5	15.5	13.8	Other
172	57	2	Survivor	0.57	5	1400	58	0	1728	14	41	4.9	67.	14.4	18.1	Other
123	57	3	Survivor	0.06	537	1206	20	0	1095	13	37	11.3	80.4	10.0	9.4	Other
174	58	1	Survivor	0.22	38	1680	80	476	702	15	45	3.6	68.3	8.4	23.2	Other
175	58	2	Survivor	0.20	50	1467	84	618	667	15	45	3.9	57.6	9.6	32.6	Other
176	58	3	Survivor	0.20	38	979	69	410	392	14	44	4.0	56.7	11.0	32.1	Other
177	59	1	Survivor	0.81	90	676	174	244	2043	10	31	10.6	80.6	7.5	11.8	Other
178	59	2	Survivor	0.64	50	654	130	201	1774	10	31	7.2	71.1	11.3	17.5	Other
179	59	3	Survivor	0.40	45	821	11	210	2017	9	28	5.7	59.5	10.8	29.5	Other
180	60	1	Survivor	0.10	38	473	73	220	963	14	45	6.0	66.1	14.1	19.6	Other
181	60	2	Survivor	0.11	13	467	74	208	975	15	46	4.4	72.2	14.9	12.8	Other
182	60	3	Survivor	0.11	17	537	82	202	989	15	47	5.8	64.9	16.5	18.4	Other
183	61	1	Survivor	0.10	19	967	13	273	816	1436	43	5.5	68.3	11.9	19.7	Other
184	61	2	Survivor	0.15	5	760	16	264	737	14	42	5.8	65.4	11.9	22.5	Other
185	61	3	Survivor	0.00	5	614	12	28	939	15	45	5.7	60.4	12.6	26.8	Other
186	62	1	Survivor	0.06	7	69	60	489	747	10	36	4.9	64.0	12.5	23.4	Other
187	62	2	Survivor	0.06	9	51	28	308	503	11	36	5.7	63.2	12.5	24.2	Other
188	62	3	Survivor	0.06	6	34	18	276	335	11	37	8.8	74.2	9.2	16.4	Other
189	64	1	Survivor	0.06	3	357	15	173	711	15	46	5.7	84.4	14.4	1.	I
190	64	2	Survivor	0.06	3	355	9	180	636	15	44	6.7	83.8	14.6	1.5	I
191	64	3	Survivor	0.06	2	290	8	157	481	14	42	4.1	62.0	17.1	20.7	Other
192	65	1	Survivor	0.23	61	382	67	445	672	15	44	4.0	80.8	5.8	13.3	Other
193	65	2	Survivor	0.28	17	402	68	462	446	14	43	6.3	75.4	11.3	13.2	Other
194	65	3	Survivor	0.22	14	496	34	628	463	14	44	5.3	75.0	13.1	11.8	Other
195	66	1	Survivor	0.11	38	473	73	207	963	14	45	6.0	66.3	14.1	19.5	Other
196	66	2	Survivor	0.11	11	467	74	203	975	15	46	4.4	72.2	14.9	12.8	Other
197	66	3	Survivor	0.06	13	537	85	220	989	15	47	5.8	64.9	16.5	18.4	Other
198	67	1	Survivor	0.09	62	747	116	282	793	12	38	6.8	77.3	9.4	13.1	Other
199	67	2	Survivor	0.09	34	747	110	284	642	12	37	6.8	74.2	9.4	16.3	Other
200	67	3	Survivor	0.06	24	525	105	358	613	12	36	7.1	71.2	9.7	18.9	Other

Table S1 - cont'd

Obs.	ID #	Day	Outcome	PCT	IL-6	Ferrit	C-RP	LDH	D-Dimer	Hb	Hemat	WBC	Neutrophil	Monocyte	Lymphocyte	Protective profiles
201	68	1	Survivor	0.18	84	164	49	611	2426	11	35	6.1	81.5	8.7	9.7	Other
202	68	2	Survivor	0.26	148	155	105	607	2195	13	39	6.1	77.2	9.1	13.5	Other
203	68	3	Survivor	0.40	215	125	51	245	3406	11	34	5.2	79.3	9.3	11.2	Other
204	69	1	Survivor	0.41	18	372	199	343	338	13	41	7.4	77.8	10.1	12.0	Other
205	69	2	Survivor	0.59	5	360	193	304	241	13	39	7.6	89.9	4.8	5.2	Other
206	69	3	Survivor	0.06	5	61	126	228	289	12	38	8.0	79.8	10.4	9.7	Other
207	70	1	Survivor	0.10	76	1616	131	292	1486	13	40	4.0	76.9	5.4	17.6	Other
208	70	2	Survivor	0.09	71	1988	133	363	1483	13	43	5.1	64.9	7.3	27.6	Other
209	70	3	Survivor	0.08	18	1303	56	434	566	13	41	6.9	74.1	7.1	18.6	Other
210	71	1	Survivor	0.12	32	1034	90	268	647	13	37	5.3	72.8	4.9	22.1	Other
211	71	2	Survivor	0.10	3	789	84	322	283	12	36	7.3	61.2	8.9	29.8	Other
212	71	3	Survivor	0.06	3	1372	62	251	220	12	37	8.6	58.5	10.1	31.2	Other
213	72	1	Survivor	0.08	11	80	22	179	784	9	29	4.0	64.6	9.1	26.2	Other
214	72	2	Survivor	0.06	7	80	14	171	1001	9	28	4.4	63.0	10.1	26.8	Other
215	72	3	Survivor	0.06	6	71	13	179	1057	8	27	4.9	60.5	9.1	30.3	Other
216	73	1	Survivor	0.24	67	260	95	292	633	12	36	8.7	78.0	6.4	15.5	Other
217	73	2	Survivor	0.21	67	360	115	416	535	13	40	8.8	80.7	3.0	16.1	Other
218	73	3	Survivor	0.13	30	358	54	286	475	13	40	10.3	73.9	11.3	14.6	Other
219	74	1	Survivor	0.08	39	93	231	148	473	11	36	5.9	55.0	10.2	34.7	Other
220	74	2	Survivor	0.06	19	88	199	212	500	11	35	6.3	52.7	8.3	38.8	III
221	74	3	Survivor	0.06	15	83	97	141	559	11	35	6.9	51.7	8.6	39.6	III
222	75	1	Survivor	0.06	20	219	21	253	796	11	35	3.6	64.7	7.2	27.9	Other
223	75	2	Survivor	0.06	31	219	37	249	784	12	39	4.3	61.5	7.5	30.8	Other
224	75	3	Survivor	0.06	18	200	44	265	774	12	36	4.0	62.7	7.6	29.5	Other
225	76	1	Survivor	0.28	233	702	22	177	1486	15	45	12.0	79.8	10.2	9.8	Other
226	76	2	Survivor	0.28	200	830	24	212	1794	13	39	7.2	79.0	7.4	13.4	Other
227	76	3	Survivor	1.44	200	2094	33	248	3508	12	35	5.3	68.1	12.9	18.8	Other
228	77	1	Survivor	0.06	5	411	41	202	1287	15	44	4.6	74.6	7.2	18.0	Other
229	78	1	Survivor	0.09	13	759	55	273	405	15	45	7.3	82.9	7.7	9.3	Other
230	78	2	Survivor	0.08	13	862	53	316	344	15	44	8.6	79.4	10.1	10.4	Other
231	78	3	Survivor	0.06	2	743	42	246	395	15	43	8.9	77.3	9.6	13.0	Other
232	79	1	Survivor	1.22	88	1427	136	477	746	11	33	6.1	76.7	9.5	13.7	Other
233	79	2	Survivor	1.73	88	1484	122	426	526	11	34	8.9	82.5	7.5	9.9	Other
234	79	3	Survivor	1.05	6	1141	85	655	382	11	36	8.4	77.7	8.6	13.5	Other
235	80	1	Survivor	0.13	29	298	67	170	869	13	40	9.0	69.7	7.2	23.0	Other
236	80	2	Survivor	0.13	29	298	6	170	888	13	40	6.9	62.0	8.8	29.1	Other
237	80	3	Survivor	0.06	3	331	77	168	432	13	40	6.8	67.4	9.3	23.2	Other
238	81	1	Survivor	0.08	8	306	3	190	294	14	42	9.1	46.0	11.0	42.8	III
239	81	2	Survivor	0.06	5	324	3	189	258	14	41	9.1	49.0	9.7	41.1	III
230	81	3	Survivor	0.06	7	417	3	198	220	14	41	9.2	50.4	9.7	39.8	III
241	82	1	Survivor	0.11	852	2923	72	509	2962	14	43	12.2	86.8	6.8	6.3	Other
242	82	2	Survivor	0.10	297	2273	69	417	1407	13	39	9.4	84.9	8.8	6.2	Other
243	82	3	Survivor	0.09	1480	1724	60	427	1402	13	41	11.9	86.2	5.4	8.2	Other
244	83	1	Non-survivor	0.15	59	612	89	256	667	11	37	6.2	70.6	7.5	21.7	Other
245	83	2	Non-survivor	0.14	11	684	94	272	515	10	33	3.8	60.5	7.4	32.0	Other
246	83	3	Non-survivor	0.25	35	512	41	362	326	12	38	4.2	69.3	6.5	24.0	Other
247	84	1	Non-survivor	0.16	16	420	16	432	707	12	37	2.9	85.8	6.9	7.2	Other
248	84	2	Non-survivor	0.11	9	721	9	628	544	13	40	3.9	85.7	8.2	5.9	Other
249	84	3	Non-survivor	0.07	19	616	6	689	758	13	40	4.2	88.2	5.7	6.0	Other
250	85	1	Survivor	0.47	73	313	188	549	954	14	42	6.0	90.5	2.0	7.4	II

Table S1 - cont’d

Obs.	ID #	Day	Outcome	PCT	IL-6	Ferrit	C-RP	LDH	D-Dimer	Hb	Hemat	WBC	Neutrophil	Monocyte	Lymphocyte	Protective profiles
251	85	2	Survivor	0.25	73	271	181	514	1204	14	40	4.0	89.5	2.7	7.7	Other
252	85	3	Survivor	0.25	52	194	42	370	696	13	37	5.3	81.5	6.7	11.7	Other
253	86	1	Survivor	0.07	3	368	46	424	337	13	40	5.3	58.6	8.0	33.2	Other
254	86	2	Survivor	0.06	3	291	52	293	313	13	40	4.6	73.9	5.4	20.5	Other
255	86	3	Survivor	0.00	3	207	71	200	341	12	39	6.5	48.3	10.1	41.4	III
256	87	1	Survivor	0.06	10	526	55	222	*	13	39	6.2	81.9	8.7	9.2	Other
257	87	2	Survivor	0.06	9	423	70	220	*	13	39	5.5	83.4	2.8	13.6	Other
258	87	3	Survivor	0.06	0	451	61	220	*	13	40	5.0	73.5	1.2	25.2	II
259	88	1	Survivor	0.36	67	4157	193	424	2767	9	29	7.9	93.4	2.2	4.3	II
260	88	2	Survivor	0.44	7	2101	125	395	14595	10	32	7.2	96.6	1.1	2.2	II
261	88	3	Survivor	0.44	5	991	31	223	1278	8	27	2.2	93.1	2.7	4.1	Other
262	89	1	Non-survivor	0.07	*	*	6	*	*	10	33	6.5	68.9	10.1	20.9	Other
263	90	1	Non-survivor	0.80	*	2678	116	*	964	14	35	6.5	75.7	12.8	11.4	Other
264	90	2	Non-survivor	0.88	*	3800	257	*	1227	13	41	3.7	76.6	9.3	14.0	Other
265	90	3	Non-survivor	0.80	*	2876	300	*	2647	11	36	3.7	69.7	9.0	21.1	Other
266	91	1	Survivor	0.16	*	*	59	*	*	12	42	4.1	54.4	10.1	35.4	Other
267	91	2	Survivor	0.19	*	*	13	*	*	12	42	4.7	58.4	9.4	32.0	Other
268	91	3	Survivor	0.12	*	*	8	*	*	11	40	5.2	64.7	8.4	26.8	Other
269	92	1	Survivor	*	*	140	3	*	*	16	48	4.4	60.9	20.4	18.6	Other
270	93	1	Non-survivor	0.21	152	487	37	561	870	12	38	3.1	72.3	8.0	19.5	Other
271	94	1	Survivor	0.06	*	*	57	*	*	14	42	4.3	43.4	20.0	36.4	III
272	94	2	Survivor	0.06	*	*	88	*	*	13	38	3.9	59.3	11.7	28.8	Other
273	94	3	Survivor	0.06	*	*	60	*	*	13	37	4.0	49.1	17.9	32.8	III
274	95	1	Survivor	*	0	202	1	*	*	14	42	6.3	59.9	14.0	26.0	Other
275	95	2	Survivor	*	*	*	*	*	*	12	37	5.5	61.6	11.4	26.8	Other
276	96	1	Survivor	0.15	29	524	131	*	*	15	48	4.3	78.3	9.5	12.0	Other
277	96	2	Survivor	0.14	58	434	160	*	*	15	48	5.0	78.0	9.9	11.9	Other
278	96	3	Survivor	*	*	420	158	*	*	15	48	5.4	78.0	9.5	12.3	Other
279	97	1	Survivor	*	*	*	*	*	*	12	35	4.8	71.4	8.6	19.9	Other
280	98	1	Survivor	*	*	*	*	*	*	13	40	6.2	54.0	13.7	32.1	Other
281	98	2	Survivor	*	*	*	*	*	*	13	39	6.7	52.70	11.9	35.3	Other
282	99	1	Survivor	*	*	*	*	*	*	12	39	7.1	66.9	6.6	26.4	Other
283	100	1	Survivor	0.13	29	298	77	477	423	13	41	8.6	89.1	1.9	8.8	II
284	100	2	Survivor	0.13	29	331	102	648	522	14	41	7.7	88.4	3.5	7.9	Other
285	100	3	Survivor	0.00	20	404	103	691	477	13	39	7.4	88.6	2.3	8.9	Other
286	101	1	Survivor	0.06	6	263	32	239	865	14	43	6.5	66.5	7.5	25.8	Other
287	101	2	Survivor	0.06	4	263	19	206	286	14	41	8.9	78.8	7.9	13.1	Other
288	101	3	Survivor	0.06	9	164	12	199	155	13	39	5.9	78.4	8.2	13.2	Other
289	103	1	Survivor	0.71	170	4231	110	632	1253	12	36	6.2	89.1	1.9	8.8	II
290	103	2	Survivor	0.42	170	4199	83	523	1407	11	34	5.5	88.5	3.5	7.9	Other
291	103	3	Survivor	0.14	223	3846	61	632	1354	12	34	8.5	88.6	2.3	8.9	Other

Table S2

**Table S2**. Temporal assessments of L-shaped distributions					
**A-** Day 1 assessment of simple and complex indicators (n=101)					
**Variable**	**Outcome***	**Count**	**Mean**	**Median**	**Kurtosis**
Lymphocyte %	NS	13	12.53	12.07	-1.19
Lymphocyte %	S	88	17.19	16.32	0.13
Neutrophil %	NS	13	81.05	81.14	-1.14
Neutrophil %	S	88	74.36	76.76	0.08
Monocyte %	NS	13	6.421	6.405	0.49
Monocyte %	S	88	8.451	7.893	0.68
LDH	NS	13	528.9	432.0	1.30
LDH	S	88	336.2	305.0	-0.08
PCT	NS	13	0.375	0.210	0.73
PCT	S	88	0.637	0.130	72.67
Ferritin	NS	13	1077	644	-0.10
Ferritin	S	88	673.4	459.0	8.90
Hb	NS	13	12.762	12.600	0.20
Hb	S	88	29.5	13.5	87.97
MCH	NS	13	30.638	30.200	2.72
MCH	S	88	29.991	29.600	68.55
RDW CV	NS	13	13.831	14.000	-0.52
RDW CV	S	88	13.217	13.000	22.08
Hematocrit	NS	13	38.12	37.30	0.36
Hematocrit	S	88	44.00	41.50	84.75
IL-6	NS	13	168.9	97.0	-0.29
IL-6	S	88	121.6	38.0	61.75
AAT	NS	13	0.02376	0.01365	-0.54
AAT	S	88	0.0680	0.0320	16.89
BBS	NS	13	2233	752	8.64
BBS	S	88	1252	177	25.24

**Table S2 - cont'd**					
**B.** Longitudinal assessment of simple and complex indicators (n=291)					
**Variable**	**Outcome***	**Count**	**Mean**	**Median**	**Kurtosis**
Lymphocyte %	NS	37	12.61	12.30	0.76
Lymphocyte %	S	254	18.454	17.215	-0.36
Neutrophil %	NS	37	81.45	82.51	-0.21
Neutrophil %	S	254	72.981	74.475	-0.56
Monocyte %	NS	37	5.938	5.332	0.01
Monocyte %	S	254	8.565	8.270	1.22
LDH	NS	37	552.4	459.0	-0.58
LDH	S	254	330.16	303.00	0.51
PCT	NS	37	0.4398	0.20	2.29
PCT	S	254	0.506	0.13	89.01
Ferritin	NS	37	1101	690	0.58
Ferritin	S	254	660.2	465.5	8.67
Hb	NS	37	12.392	12.200	0.08
Hb	S	254	18.57	13.30	253.78
MCH	NS	37	30.584	30.200	1.24
MCH	S	254	29.913	29.450	57.89
RDW CV	NS	37	13.889	14.000	-0.91
RDW CV	S	254	13.304	13.100	21.68
Hematocrit	NS	37	37.424	37.300	0.42
Hematocrit	S	254	40.65	40.10	223.02
IL-6	NS	37	410	66	12.43
IL-6	S	254	103.8	25	74.27
AAT	NS	37	0.02360	0.01328	0.48
AAT	S	254	0.07276	0.03681	13.41
BBS	NS	37	3213	752	7.35
BBS	S	254	1455	144	184.52

**Table S2 - cont'd**					
**C**. Dynamics-related kurtosis (day 2 minus day 1 values, n=92 pairs)					
**Variable**	**Outcome***	**Count**	**Mean**	**Median**	**Kurtosis**
Net d2-d1 L %	NS	11	2.72	1.27	1.84
Net d2-d1 L %	S	81	2.277	1.604	3.58
Net d2-d1 N %	NS	11	-2.51	-0.10	1.69
Net d2-d1 N %	S	81	-2.284	-1.375	1.74
Net d2-d1 M %	NS	11	-0.210	-0.121	0.13
Net d2-d1 M %	S	81	0.007	0.207	1.52
Net d2-d1PCT	NS	11	0.074	-0.010	3.10
Net d2-d1PCT	S	81	-0.144	0.000	41.35
Net d2-d1 Hb	NS	11	-0.555	-0.600	-0.44
Net d2-d1 Hb	S	81	-18.0	-0.5	80.99
Net d2-d1 Hematocrit	NS	11	-0.83	-1.7	-0.90
Net d2-d1 Hematocrit	S	81	-5.31	-1.0	80.08
Net d2-d1 IL-6	NS	10	38.4	0	8.37
Net d2-d1 IL-6	S	81	-27.5	0	34.41
Net d2-d1*AAT*	NS	11	0.00780	0.00276	3.39
Net d2-d1*AAT*	S	81	0.00567	0.00255	18.37
Net d2-d1*BBF*	NS	11	0.00647	0.00000	4.81
Net d2-d1*BBF*	S	81	0.00376	0.00036	19.14
Net d2-d1*BBT*	NS	11	0.000773	0.000015	4.34
Net d2-d1*BBT*	S	81	-0.00019	0.00002	29.69
Net d2-d1*BAS*	NS	11	3.1	-12.6	7.87
Net d2-d1*BAS*	S	81	-3.5	-2.0	35.84
Net d2-d1[L/M]/ [N/L]	NS	11	.344	.044	2.5
Net d2-d1[L/M]/ [N/L]	S	81	.271	.095	24.65
